# Risk Factors for Dementia Incidence Based on Previous Results of the Specific Health Checkups in Japan

**DOI:** 10.3390/healthcare8040491

**Published:** 2020-11-17

**Authors:** Yoh Tamaki, Yoshimune Hiratsuka, Toshiro Kumakawa

**Affiliations:** 1 Department of Health and Welfare Services, National Institute of Public Health, Wako, Saitama 351-0197, Japan; yoshi-h@tkf.att.ne.jp (Y.H.); kumakawa.t.aa@niph.go.jp (T.K.); 2 Department of Ophthalmology, Juntendo University School of Medicine, Tokyo 113-8431, Japan; 3 The University of Fukuchiyama, Kyoto 620-0886, Japan

**Keywords:** dementia, primary care, long-term care, risk factor, checkups, abdominal circumference

## Abstract

Dementia is a common disease in elderly people, with its prevalence expanding rapidly worldwide. Longitudinal and cohort studies on lifestyle and health conditions are needed to identify the risk of dementia. This study aimed to identify the risk factors for dementia incidence in Japan and to clarify the strategy for its primary care. In this study, an analysis was performed to investigate the association between the cognitive faculty level of the long-term care certification survey and the previous results of the specific health checkups in Japan. To investigate the risk factor for dementia incidence, a multivariable logistic regression analysis was performed, which showed a significant odds ratio for the incidence of dementia for two items, including abdominal circumference and insulin injections or oral hypoglycemic medications. The findings of our study suggested that a lower abdominal circumference had a higher risk for dementia incidence, and individuals who received insulin injections or oral hypoglycemic medications had a higher risk for dementia incidence based on the results of the health checkups conducted 10 years previously. Further, longer duration study with a larger sample is needed to identify the items from the specific health checkups that are associated with the risk of dementia.

## 1. Introduction

Dementia is a common disease in elderly people, with its prevalence expanding rapidly worldwide [1]. The World Health Organization (WHO) reported that the number of dementia patients is estimated to be 36 million in 2012 [2]. This report estimates that the number of people with dementia will double every 20 years, reaching 66 million by 2030 and 115 million by 2050 [3]. In 2013, the Ministry of Health, Labor, and Welfare research group reported 4.62 million dementia patients in Japan, increasing to 6.75 million by 2025 and 8.20 million by 2030 [4]. 

The risk of dementia increases with age, especially showing rapid growth in individuals aged 70 and above. The onset of dementia imposes a heavy burden on the patient himself/herself and on family life. Moreover, from the perspective of social security costs, concrete and standard preventive measures for dementia should be urgently established. It is necessary to clarify the risk factors for dementia to establish preventive methods. Generally, preventive methods are assigned to three stages: “primary prevention,” “secondary prevention,” and “tertiary prevention.” From the perspective of preventing the development of dementia, “primary prevention” and “secondary prevention” are especially important. Longitudinal and cohort studies on lifestyle and health conditions are needed to identify the risk of dementia. As lifestyle-related diseases, middle-aged metabolic syndrome and elderly diabetes are listed as dementia risks. Ng et al. reported that the metabolic syndrome was associated with an increased incidence of cognitive impairment and progression to dementia [5]. Furthermore, diabetes is increasingly recognized as an important risk factor of cognitive function [6,7,8,9,10,11]. However, the association of higher obesity in mid-age with the incidence of dementia is debatable. Albanese et al. in their systematic review reported increased risk of dementia due to obesity in midlife, although the association between being underweight and dementia was disputable [12]. Pedditzi et al., in their meta-analysis, suggested a positive association between obesity in midlife and later dementia, and further studies are required to assess whether weight reduction in midlife reduces risk [13].

Weight loss associated with dementia begins before the onset of the clinical syndrome and accelerates by the time of diagnosis [14]. Epidemiological evidence suggests that dementia may cause involuntary weight loss long before its clinical onset, and low BMI can appear to be detrimental to dementia [15,16,17]. However, many factors, such as depression, diabetes, hypertension, and stroke may confound or mediate the association between BMI and dementia, and the covariates of statistical models have varied significantly between previous studies [13]. Therefore, in order to eliminate these confounding factors, it is necessary to perform multivariate analysis including data such as various biochemical test values and medical history. In Japan, Yokomichi reported that underweight and diabetes were risk factors for dementia incidence and lower body mass index (BMI) was also associated with dementia incidence in elderly adults [18]. Therefore, it is critical to analyze the correlation between the incidence of dementia and being underweight by ethnic group and food culture [18]. 

From 2008, the Ministry of Health, Labor, and Welfare in Japan started a standard health checkup program to identify people with high risk for metabolic syndrome. The Japanese health checkup/guidance program is a health policy to prevent metabolic syndrome and reduce social security costs in the medium and long term through lifestyle interventions [19]. The specified health checkups in Japan comprise 30 items, including a physical examination to assess risk factors for metabolic syndrome, and a 23-item questionnaire. In addition, Japanese long-term care insurance is a compulsory insurance that people above the age of 40 are obliged to join. A variety of care services are provided to residents who need nursing care at cut rates [20]. For this insurance, residents over the age of 65 years (category 1) and those from 40 to 64 years (category 2) are covered. Long-term care insurance services are provided if an individual over the age of 65 needs care or support, or if those between 40 and 65 years develop an age-related disease requiring care or support [21]. In the certification process for long-term care in Japan, the Needs Certification Committee conducts a survey of a total of 74 items, including physical function, cognitive function, life function, daily activity function, behavioral disorder, and adjustment to social life. The required level of care is classified into a total of 7 levels (support needs levels 1–2 and care needs levels 1–5) [22]. The data from the care certification survey include items associated with dementia. Cognitive function comprises seven stages of daily life.

In this study, an analysis was performed to investigate the association between the cognitive faculty level of the long-term care certification survey and the previous results of the specific health checkups in Japan. This study aimed to identify the risk factors for dementia incidence in Japan and to clarify the strategy for its primary care.

## 2. Materials and Methods

### 2.1. Study Population

The subjects investigated in this study were selected from 30,757 insured residents in Mishima City, Shizuoka, Japan (total population 107,000). A total of 606 persons (207 men and 399 women; average age 79.5 ± 4.27 years, range 55–84 years) who submitted to both long-term care insurance services in 2018 and a previous standard program of health checkups in 2008 were included in this study.

The data of long-term care certification were analyzed to identify the association between the cognitive faculty level in 2018 and the previous results of the specific health checkups in 2008.

In the long-term care certification process, the cognitive faculty level was categorized by the long-term care support specialists into a total of seven degrees of independence in daily living (I: can do on their own, IIa: monitoring needed only outside home, IIb: monitoring needed inside and outside home, IIIa: need support during day, IIIb: need support during night, IV: need support during day and night, and M: need support in nursing home). The checkup programs in this study were offered to community insured between 40 and 65 years in 2008. The checkup comprised 30 items, including physical and laboratory examination.

### 2.2. Statistical Analysis

Factors affecting dementia incidence were assessed using logistic regression analysis. To investigate the risk factors for dementia incidence, a multiple logistic regression analysis was simultaneously carried out to calculate the adjusted odds ratio and exclude the confounding effects. Before analysis, the patients were classified into two groups: (I, IIa, IIb) and (IIIa, IIIb, IV, M). A multivariable logistic regression analysis was performed with the cognitive faculty level as the objective variable (III–M/I–IIb) and previous results of the specific health checkups (34 items associated with age, sex laboratory test, physical examination, drug-taking behavior, and outpatient medical expenditures) as the explanatory variable. The statistical analyses described above were performed using SPSS ver. 25 and Modeler ver. 18.1 (IBM Japan Ltd., Tokyo, Japan).

This study was approved by the ethics committee of the National Institute of Public Health (NIPH-IBRA #12137) and the municipal assembly of Mishima. All procedures were conducted according to the International Ethical Guidelines for Epidemiology [23], Guidelines for the Utilization of the Database for National Health Insurance Claim, Specific Medical Checkup/Health Guidance [24], and Guidelines of Security for Health Information Systems [25].

Following the guidelines above, the researchers conducted analyses after the personal data were anonymized by the local administration.

## 3. Results

Table 1 shows the degree of dementia in daily living used in long-term care certification. 

**Figure 1:**
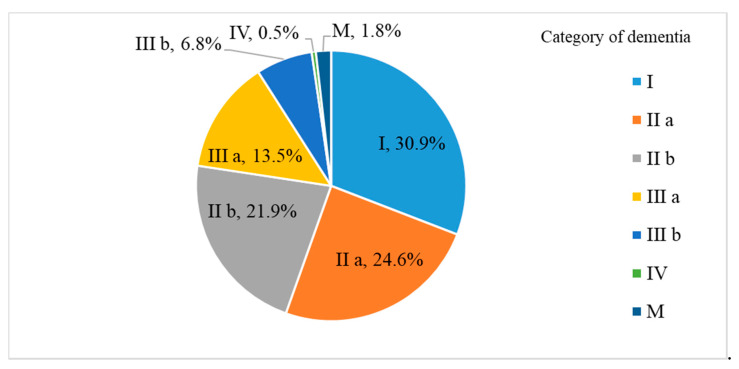
Results of degree of dementia in daily living (2018).

The descriptive statistics of the degree of dementia in daily living (2018) are shown in Figure 1.

About 22.6% of individuals above category IIIa needed support in daily living. Table 2 shows the descriptive statistics of the biochemical examinations conducted in 2008. 

**Figure 2:**
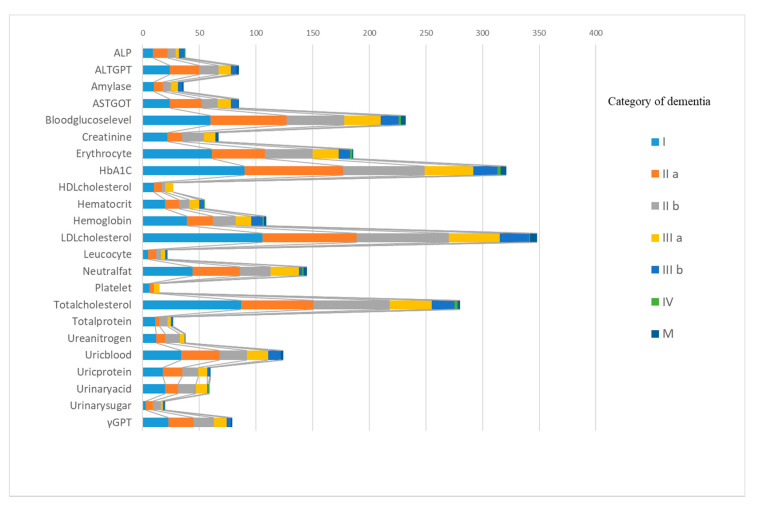
The number of abnormal subjects for each item by the category of dementia.

The percentages above the category of follow-up were higher for low-density-lipoprotein cholesterol, total cholesterol, and glycosylated hemoglobin than for other items. Figure 2 shows the number of abnormal subjects (above the category of follow-up) for each item by the category of dementia.

To investigate the risk factor for dementia incidence, a multivariable logistic regression analysis was performed, which showed a significant odds ratio (OR) for the incidence of dementia for two items, including abdominal circumference (cm) and insulin injections or oral hypoglycemic medications (Yes/No) (Table 3). A higher abdominal circumference had a lower risk for dementia incidence (OR: 0.961, 95% CI: 0.927–0.997). Individuals who received insulin injections or oral hypoglycemic medications had a higher risk of dementia incidence than those who did not (OR: 2.635, 95% CI: 1.294–5.365). No significant OR was obtained for other biochemical examinations, physical examinations, and outpatient medical expenditures in 2008.

## 4. Discussion

The number of beneficiaries and eligible persons in Japan requiring long-term care under this insurance system increased from 2.2 million in 2000 to 6.6 million in 2019. About one-fifth of Japanese people above the age of 65 years are certified for long-term care [21]. In Japan, to receive long-term care services, it is mandatory to apply for the required nursing care certification and receive it from the municipality where an individual stays. In the long-term care survey, healthcare professionals assess the physical and mental status of an applicant and determine the level of care required. The results of this survey are used to determine the required care level for all applicants in Japan. The assessment data collected by this procedure are expected to be aggregated at the national level and stored in a large database. This database could be useful to identify the risk factors for dementia incidence to clarify the strategy for its primary care.

Moreover, in Japan over ten years have passed since the specific medical checkup and health guidance started from 2008 [26]. A database of the National Health Insurance Claim for specific medical checkups and specific health guidance shows the difference in annual medical expenses between patients classified with and without metabolic syndrome to use in a probabilistic way to determine regional medical costs [27]. The results of these medical checkups screen people who need specific health guidance by the risk of metabolic syndrome and assign them to three levels of support (I: information service only, II: motivational support, III: active support). Those who have undergone a specific medical examination are notified of the necessary information and the need for guidance based on the examination results. The specific healthcare guidance supports the improvement of lifestyle habits, such as nutrition and exercise, according to the risk of lifestyle-related diseases. People classified as motivational and active support are offered initial counseling and a final assessment six months later. If risk factors for dementia incidence based on previous results of the specific health checkups could be identified, these guidances could provide assistance to reduce the risk of dementia with the improvement of lifestyle and by establishing preventive methods. In this study, by matching the database of specific medical checkups with long-term care certification, the risk factors for dementia incidence were identified.

The results of this study showed that a lower abdominal circumference had a higher risk of dementia. Qizilbash et al. reported that being underweight in middle and old age increased the risk of dementia for two decades [28]. Nam et al. reported that a lower baseline BMI was related with increased risks of all-cause dementia, and weight loss was related with an increased risk of dementia [29]. However, Kivimäki et al. reported that high BMI was associated with an increased risk of dementia when BMI was assessed more than 20 years before the diagnosis of dementia. Conversely, low BMI was reported to be a predictor of dementia if the assessment was performed less than 10 years before diagnosis [30]. In Japan, Yokomichi reported that underweight men with dyslipidemia and underweight women with hypertension had a higher risk for dementia [14]. The results of our study were consistent with those of Yokomichi’s study. However, because these two studies in Japan had a research period of 10 years or less, further long-term studies are needed.

These previous studies used body weight or BMI as explanatory variables. Unlike these previous studies, this study added abdominal circumference as an explanatory variable along with BMI. As a result, no significant odds ratio was obtained for BMI, but a significant odds ratio was obtained for abdominal circumference. The abdominal circumference is usually considered as an index of the amount of visceral fat, but there are few studies on the relationship between the abdominal circumference and dementia incidence. Further studies using the abdominal circumference as an explanatory variable are needed in the future.

In this study, we found that individuals who received insulin injections or oral hypoglycemic medications had a higher risk of dementia incidence. Previous studies have confirmed the progression of hippocampal atrophy in diabetic patients with a long duration of illness, with diabetes reported as a risk factor for dementia in Japan [31,32,33].

In the future, it will be necessary to clarify the contribution rate with higher accuracy and the risk of lifestyle-related diseases, such as diabetes, for the development of dementia in Japan. In such a scenario, the specific health checkups and the specific healthcare guidance currently being conducted for the prevention of metabolic syndrome, may be possibly used for the prevention of dementia. Thus, further, longer duration study with a larger sample is needed to identify the items from the specific health checkups that are associated with the risk of dementia.

## 5. Conclusions

The findings of our study suggested that a lower abdominal circumference had a higher risk for dementia incidence, and individuals who received insulin injections or oral hypoglycemic medications had a higher risk for dementia incidence based on the results of the health checkups conducted 10 years previously. Further, longer duration study with a larger sample is needed to identify the items from the specific health checkups that are associated with the risk of dementia.

## Figures and Tables

**Table 1:** Degree of dementia in daily living.

Category	Degree of Independence in Daily Living
Ⅰ	Can do on their own
Ⅱa	Monitoring needed only outside home
Ⅱb	Monitoring needed inside and outside home
Ⅲa	Need support during day
Ⅲb	Need support during night
IV	Need support during day and night
M	Need support in nursing home

**Table 2:** Results of biochemical examination (2008).

Item	Normal	Follow-Up	Requires Further Testing	Requires Treatment	Total
ALP	568	38	0	0	606
	93.7%	6.3%	0.0%	0.0%	100.0%
ALT(GPT)	521	68	0	17	606
	86.0%	11.2%	0.0%	2.8%	100.0%
Amylase	570	36	0	0	606
	94.1%	5.9%	0.0%	0.0%	100.0%
AST(GOT)	521	71	0	14	606
	86.0%	11.7%	0.0%	2.3%	100.0%
Blood glucose level	374	161	0	71	606
	61.7%	26.6%	0.0%	11.7%	100.0%
Creatinine	539	67	0	0	606
	88.9%	11.1%	0.0%	0.0%	100.0%
Erythrocyte	420	165	0	21	606
	69.3%	27.2%	0.0%	3.5%	100.0%
HbA1C	285	237	0	84	606
	47.0%	39.1%	0.0%	13.9%	100.0%
HDL-cholesterol	579	20	0	7	606
	95.5%	3.3%	0.0%	1.2%	100.0%
Hematocrit	550	50	0	5	606
	90.9%	8.3%	0.0%	0.8%	100.0%
Hemoglobin	496	82	0	27	606
	82.0%	13.5%	0.0%	4.5%	100.0%
LDL-cholesterol	258	348	0	0	606
	42.6%	57.4%	0.0%	0.0%	100.0%
Leucocyte	584	22	0	0	606
	96.4%	3.6%	0.0%	0.0%	100.0%
Neutral fat	461	126	0	19	606
	76.1%	20.8%	0.0%	3.1%	100.0%
Platelet	590	15	0	0	606
	97.5%	2.5%	0.0%	0.0%	100.0%
Total protein	579	27	0	0	606
	95.5%	4.5%	0.0%	0.0%	100.0%
Total-cholesterol	326	237	0	0	606
	53.8%	39.1%	0.0%	0.0%	100.0%
Urea nitrogen	568	38	0	0	606
	93.7%	6.3%	0.0%	0.0%	100.0%
Uric blood	482	14	92	18	606
	79.5%	2.3%	15.2%	3.0%	100.0%
Uric protein	546	3	38	19	606
	90.1%	0.5%	6.3%	3.1%	100.0%
Urinary acid	547	59	0	0	606
	90.3%	9.7%	0.0%	0.0%	100.0%
Urinary sugar	586	0	6	14	606
	96.7%	0.0%	1.0%	2.3%	100.0%
γGTP	527	50	0	29	606
	87.0%	8.3%	0.0%	4.8%	100.0%

**Table 3:** Results of multivariate logistic regression analysis.

Item	Multivariate Adjusted Odds Ratio	95% CI		*p*-Value
		Lower Limit	Upper Limit	
Age	1.038	0.985	1.095	0.161
Sex (Women/Men)	0.975	0.488	1.948	0.942
Height (cm)	0.992	0.955	1.032	0.698
Abdominal circumference (cm)	0.961	0.927	0.997	0.032
BMI	1.039	0.934	1.156	0.484
Medicine to lower blood pressure (Yes/No)	0.907	0.581	1.416	0.669
Insulin injections or oral hypoglycemic medications (Yes/No)	2.635	1.294	5.365	0.008
Medicine to lower cholesterol (Yes/No)	1.001	0.630	1.590	0.998
Systolic blood pressure (mmHg)	1.007	0.990	1.025	0.396
Diastolic blood pressure (mmHg)	0.973	0.946	1.001	0.063
ALP (+±/−)	1.122	0.482	2.609	0.790
ALT(GPT) (+±/−)	1.082	0.621	1.884	0.781
Amylase (+±/−)	1.482	0.664	3.308	0.337
AST(GOT) (+±/−)	0.883	0.473	1.646	0.695
Blood glucose level (+±/−)	0.958	0.715	1.285	0.776
Creatinine (+±/−)	0.854	0.418	1.747	0.666
Erythrocyte (+±/−)	0.922	0.655	1.298	0.640
HbA1C (+±/−)	0.857	0.631	1.164	0.322
HDL-cholesterol (+±/−)	1.363	0.797	2.331	0.257
Hematocrit (+±/−)	1.467	0.763	2.821	0.250
Hemoglobin (+±/−)	0.893	0.618	1.289	0.545
LDL-cholesterol (+±/−)	1.086	0.669	1.761	0.739
Leucocyte (+±/−)	1.222	0.438	3.405	0.702
Neutral fat (+±/−)	1.150	0.795	1.664	0.458
Platelet (+±/−)	1.554	0.499	4.837	0.446
Total protein (+±/−)	0.787	0.277	2.236	0.653
Total-cholesterol (+±/−)	0.964	0.716	1.298	0.807
Urea nitrogen (+±/−)	0.549	0.193	1.561	0.261
Uric blood (+±/−)	1.130	0.897	1.423	0.301
Uric protein (+±/−)	0.940	0.684	1.290	0.701
Urinary acid (+±/−)	1.089	0.503	2.362	0.828
Urinary sugar (+±/−)	0.973	0.600	1.579	0.913
γGPT (+±/−)	1.048	0.725	1.515	0.804
Outpatient Medical Expenditures in 2008	1.000	1.000	1.000	0.609
_cons	1.087			0.985

Dependent variable was classified to (IIIa, IIIb, IV, M)/(I, IIa,IIb). A Total of 34 items were entered simultaneously as the independent variables.
